# Energy-efficient transmission strategies for CoMP downlink—overview, extension, and numerical comparison

**DOI:** 10.1186/s13638-018-1214-2

**Published:** 2018-08-20

**Authors:** Kien-Giang Nguyen, Oskari Tervo, Quang-Doanh Vu, Le-Nam Tran, Markku Juntti

**Affiliations:** 1 Centre for Wireless Communications, University of Oulu, Oulu, P.O. Box 4500, FI-90014 Finland; 2 School of Electrical and Electronic Engineering, University College Dublin, Dublin, Ireland

**Keywords:** Energy efficiency, Generalized Dinkelbach’s algorithm, Successive convex approximation, Fractional programming, Power consumption, Coordinated beamforming

## Abstract

This paper focuses on energy-efficient coordinated multi-point (CoMP) downlink in multi-antenna multi-cell wireless communications systems. We provide an overview of transmit beamforming designs for various energy efficiency (EE) metrics including maximizing the overall network EE, sum weighted EE, and fairness EE. Generally, an EE optimization problem is a nonconvex program for which finding the globally optimal solutions requires high computational effort. Consequently, several low-complexity suboptimal approaches have been proposed. Here, we sum up the main concepts of the recently proposed algorithms based on the state-of-the-art successive convex approximation (SCA) framework. Moreover, we discuss the application to the newly posted EE problems including new EE metrics and power consumption models. Furthermore, distributed implementation developed based on alternating direction method of multipliers (ADMM) for the provided solutions is also discussed. For the sake of completeness, we provide numerical comparison of the SCA based approaches and the conventional solutions developed based on parametric transformations (PTs). We also demonstrate the differences and roles of different EE objectives and power consumption models.

## Introduction

Fifth generation (5G) wireless network visions foresee the challenges of the data traffic demand caused by the upcoming explosive growth of wireless devices and applications [1]. The rapid expansion of mobile networks is increasing the energy consumption beyond sustainable limits. In the larger base stations (BSs), the most power-hungry components of the multi-antenna transmitters are the transmit power amplifiers (PAs), but the other circuits and components are also significant power consumers. In fact, they become even dominant in the smaller BSs, which are becoming more and more popular in the future dense networks. Nevertheless, this causes problems in terms of electricity costs for operators and the increase in greenhouse gas emission for the whole world [2, 3]. Consequently, energy efficiency (EE) has become an important design target for wireless access networks.

In wireless communications, energy efficiency is generally defined as the ratio of the total reliably transmitted data to the total energy consumption [4]. In other words, it equals the achievable data rate in bits per second divided by the consumed power in Watts. In either case, the basic unit of EE is bits per Joule (bits/J). It is worth mentioning that the classical transceiver optimization framework, on the other hand, typically focuses on maximizing the multi-user weighted sum rate or (area) spectral efficiency regardless of the proportionally rapid increase of total power consumed by the wireless network. The EE optimization deviates from this setup by making a controlled trade-off between the supported rate and the consumed power [2, 3, 5–7].

Variations of the EE objective have been proposed depending on the system constraints and design targets. The basic alternatives include *network EE* (NEE), *sum weighted EE* (SWEE), and *fairness EE* [5]. While the first metric optimizes the EE gain of the entire network, the others aim at satisfying the specific EE requirements on individual base stations or users involved.

In the presence of multi-user interference, an EE maximization (EEmax) problem belongs to a class of nonconvex fractional programs for which finding a globally optimal solution is challenging. However, an optimal solution of the EEmax problem in multi-user multiple-input single-output (MISO) downlink system has been provided in [7] using a branch-reduce-and-bound approach. Even though this approach guarantees finding the global optimum, it still requires very high computational complexity. Therefore, low-complexity suboptimal designs have attracted more attention for practical applications.

Common suboptimal approaches for EE designs have been developed based on parametric transformation (PT) inspired by the fractional structure of the EE objectives [5, 8, 9]. However, such an approach leads to two-layer iterative procedures [9], which often have high computational complexity (as discussed in Section 3.1) and/or are not suitable for distributed implementation. In addition, analyzing the convergence of those methods has not been properly addressed [7].

Recently, novel algorithms have been developed based on the state-of-the-art local optimization toolbox, namely successive convex approximation (SCA) algorithm, which efficiently solves the EEmax problems; the proposed framework is a one-loop iterative procedure which finds out locally optimal solutions after a relatively small number of iterations and, thus, significantly reduces the complexity compared to the existing PT approach [10]; the convergence of the SCA-based methods is provably guaranteed [7, 10], and the procedure is also well suited for the implementation in a distributed manner [11].

In this paper, we consider coordinated multi-point (CoMP) downlink in multi-antenna multi-cell systems and focus on the applications of the SCA approach on the EEmax problems arising in the wireless access systems such as 4G and 5G cellular standards. The main contributions of this paper can be summarized as follows:

- Overview: We provide a summary of the basic concepts of the SCA-based algorithms; introduce some key transformations which turn the EEmax problems into representations that successfully leverage the principle of the SCA; revisit the problems of maximizing the NEE, SWEE, and maxminEE; and discuss how to arrive at efficient solutions. We also discuss how to distributively implement the solutions.
- Extension: We discuss the recently proposed weighted product EE (WPEE) objective function and a general model of power consumption. We show how to adopt the proposed framework to the EEmax problems involved.
- Numerical comparisons: We make several numerical comparisons on the algorithms. The most important one is the comparison between the existing and the proposed approaches in terms of convergence speed and average performances. Other evaluations have been made to illustrate the roles and benefits of different EE objectives and the impact of different power consumption models on the EE performance.

An initial version of the paper was published in [12]. Herein, we provide a more detailed and broader summary of the EE optimization and discussion on the differences of the SCA- and fractional programming-based approaches. We also extend the SCA framework to solve the problem of WPEE maximization. We further present four different approximations for the involved logarithmic functions, which enable the second-order programming formulations of the problems. Finally, we consider more detailed power consumption models and provide a significantly more extensive set of simulation results to evaluate different methods.

The rest of the paper is organized as follows. System model and several energy efficiency measures are presented in Section 2. Centralized solutions and their distributed implementation are provided in Section 3, followed by numerical results in Section 4. Conclusion is provided in Section 5.

*Notation*: Bold lower and upper case letters represent vectors and matrices, respectively; calligraphic letters denote sets; |·| represents the absolute value; ∥·∥_2_ represents the *l*_2_ norm; $\mathcal {CN}(0,a)$ denotes a zero-mean circularly symmetric complex Gaussian random variable with variance *a*; $\mathbb {C}^{a\times b}$ represents the space of complex matrices of dimensions given in superscript; ${\mathfrak {R}}(\cdot)$ represents real part of the argument; $\mathbb {E}\{\cdot \}$ denotes the expectation operator. **a**^*T*^ and **a**^*H*^ stand for the transpose and the Hermitian transpose of **a**, respectively. 〈**a**,**b**〉 denotes the inner product of vectors **a** and **b**. $\{\mathbf {a}_{b}\}_{b\in \mathcal {B}}$ refers to a composite vector containing all **a**_*b*_ where *b* belongs to the set $\mathcal {B}$. ∇_**x**_*g*(**x**) represents the partial derivative of function *g*(**x**) with respect to the elements of **x**. Other notations are defined at their first appearance.

## System model and energy-efficient problem formulations

### Channel and signal model

**Fig. 1:**
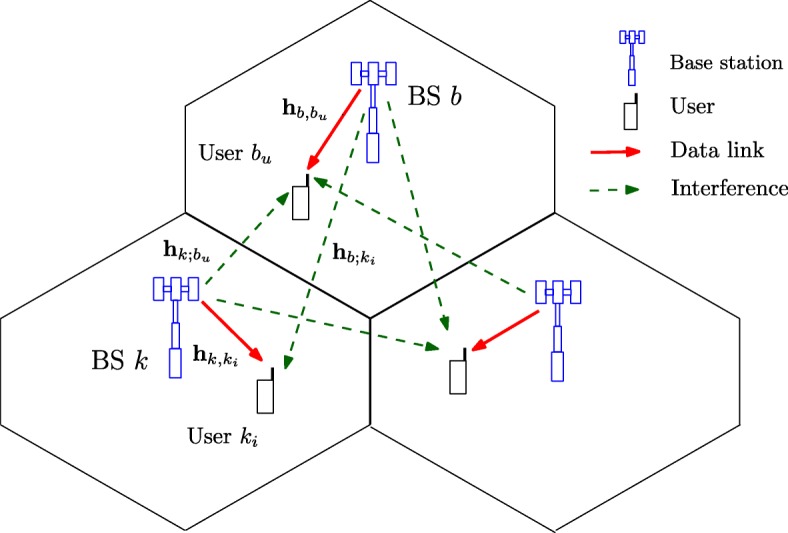
CoMP system model

We consider a downlink transmission in multi-cell multi-user multiple-input single-output (MISO) system consisting of *B* BSs, each of which is equipped with *M* antennas. There are *U* single-antenna users in each cell and a total of *UB* users in the network^1^. We assume that the BSs operate following the coordinated beamforming mode, i.e., each BS only serves *U* users in its own cell.^2^ The considered system model is illustrated in Fig. 1. The beamforming vectors are designed to control the interference between the cells so as to maximize a performance target [13]. Let us denote the set of BSs by ${\mathcal {B}}=\{1,\ldots,B\}$ and the set of users in cell *b* by ${\mathcal {U}}_{b}=\{1,\ldots,U\}$. User *u* in cell *b* is denoted compactly as *b*_*u*_. Let $s_{b_{u}}$ be an independent data symbol for user b_u_ which is assumed to have a unit energy, i.e., $\mathbb {E}\{|s_{b_{u}}|^{2}\}=1$. Linear transmit precoding is adopted such that the signal transmitted to user *b*_*u*_ (from BS *b*) is a multiplication of $s_{b_{u}}$ and transmit beamforming vector $\mathbf {v}_{b_{u}}\in \mathbb {C}^{M\times 1}$. Let $\mathbf {h}_{k,b_{u}}\in \mathbb {C}^{1\times M}$ denote the flat-fading channel (row) vector between BS *k* and user *b*_*u*_. The received signal at user *b*_*u*_ can be written as

1 $$ \begin{aligned} y_{b_{u}}= & \ \mathbf{h}_{b,b_{u}}\mathbf{v}_{b_{u}}s_{b_{u}}+ \sum_{i\in{\mathcal{U}}_{b}\backslash\{b_{u}\}}\mathbf{h}_{b,b_{u}}\mathbf{v}_{b_{i}}s_{b_{i}}+ \sum_{k\in{\mathcal{B}}\backslash\{b\}}\sum_{i\in{\mathcal{U}}_{k}}\mathbf{h}_{k,b_{u}}\mathbf{v}_{k_{i}}s_{k_{i}}+z_{b_{u}}, \end{aligned}   $$

where $z_{b_{u}}$ is the additive white Gaussian noise with distribution $z_{b_{u}}\sim {\mathcal {C}N}(0,\sigma _{b_{u}}^{2})$, $\sigma _{b_{u}}^{2}=WN_{0}$ is the noise power when using the transmission bandwidth *W*, and the noise power spectral density is *N*_0_. In (1), the second and third terms represent the intra-cell and inter-cell interference, respectively. Let us denote by $G_{b_{u}}(\mathbf {v})\triangleq \sum _{i\in {\mathcal {U}}_{b}\backslash \{b_{u}\}}|\mathbf {h}_{b,b_{u}}\mathbf {v}_{b_{i}}|^{2}+\sum _{k\in {\mathcal {B}}\backslash \{b\}}\sum _{i\in {\mathcal {U}}_{k}}|\mathbf {h}_{k,b_{u}}\mathbf {v}_{k_{i}}|^{2}$ the power of interference at user *b*_*u*_. As is common in the system optimization, we use the information theoretic rate expressions of the Gaussian channels. Those assume the use of Gaussian codebooks. Therefore, the multi-user interference terms can be modeled as additive colored Gaussian noise, and the signal-to-interference-plus-noise ratio (SINR) at user *b*_*u*_ is expressed as

2 $$ \Gamma_{b_{u}}(\mathbf{v}) \triangleq \frac{|\mathbf{h}_{b,b_{u}}\mathbf{v}_{b_{u}}|^{2}}{G_{b_{u}}(\mathbf{v})+\sigma_{b_{u}}^{2}}.  $$

The data rate of user *b*_*u*_ is given by $r_{b_{u}}(\mathbf {v})=W\log (1+\Gamma _{b_{u}}(\mathbf {v})$), and the total data rate over the network is given by

3 $$ R(\mathbf{v}) \triangleq \sum_{b\in{\mathcal{B}}}\sum_{u\in{\mathcal{U}}_{b}}r_{b_{u}}(\mathbf{v}).  $$

#### Transmit power constraints

Since the available power budget at the BSs is finite, the transmit power at each BS should satisfy

4 $$ \sum_{u\in{\mathcal{U}}_{b}}\|\mathbf{v}_{b_{u}}\|_{2}^{2}\leq P_{b},\forall b\in{\mathcal{B}},  $$

where *P*_*b*_ is the transmit power budget at BS *b*. In practice, the power amplifier at each antenna chain is designed to operate over a specific power range, i.e, the output power should not exceed a predefined threshold. Thus, the power constraint for each antenna can be also imposed, i.e.,

5 $$ \sum_{u\in{\mathcal{U}}_{b}}|[\mathbf{v}_{b_{u}}]_{m}|^{2}\leq P_{b}^{m},\forall b\in{\mathcal{B}},\ m=1,2,\ldots,M,  $$

where [**x**]_*m*_ denotes the *m*th element of vector **x**, and $P_{b}^{m}$ is the maximum transmit power at the *m*th antenna of BS *b*. Several other power constraints could be applied, but we focus on these most common ones.

### Power consumption model

**Fig. 2:**
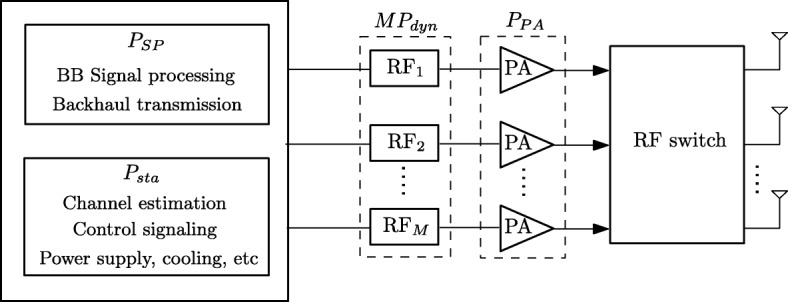
Power consumption model for a BS

The consumed power can be classified into three main categories: circuit operation power in network elements, signal processing power, and power dissipated on power amplifiers (PAs). Some of the power components are static (sta), while others are dynamic (dyn) or rate-dependent (RD). The power consumption model is sketched in Fig. 2.

#### Circuit power

A significant amount of power is used to operate the electronic circuits of network elements such as the base stations, site cooling, control signaling, backhaul infrastructure, and some parts of the baseband processors. In addition, a radio frequency (RF) chain including, for example, converters, filters, and mixers requires some operating power. In general, we can express the amount of power consumption for operating transceiver circuits in cell *b* as [5, 14, 15]

6 $$ P_{\text{cir},b} \triangleq P_{\text{sta}}+{MP}_{\text{dyn}}+{UP}_{\text{Us}},  $$

where *P*_sta_ and *P*_dyn_ represent for static and dynamic power consumption at BS *b*, respectively, and *P*_Us_ accounts for power running a user device.

#### Signal processing power

The data needs to be encoded and modulated at the transmitter as well as demodulated and decoded at the receiver. Conventionally, the amount of power for these functionalities is assumed to be fixed [5, 8, 9, 16, 17]. However, generally, a higher data rate requires a larger codebook, and the larger number of bits incurs higher power for encoding and decoding on baseband circuit boards. Moreover, the backhaul is used to transmit data between the core network and the BSs, and the power consumed for the backhaul also increases with the data rate [18–20]. From this perspective, signal processing power consumption is rate-dependent and is assumed to be a linear function of the transmission rate [18]. Let us denote by *P*_SP,*b*_(*r*_*b*_(**v**)), where $r_{b}(\mathbf {v}) \triangleq \sum _{u\in {\mathcal {U}}_{b}}r_{b_{u}}(\mathbf {v})$ is the signal processing power for BS *b*. Then, we can write

7 $$ \begin{aligned} P_{\text{SP},b}(r_{b}(\mathbf{v}))\triangleq\!\left\{\begin{array}{ll} \text{constant} & \textrm{for\ fixed\ power\ model}\\ {p_{\text{SP}}\sum\limits_{u\in{\mathcal {U}}_{b}}r_{b_{u}}(\mathbf{v})} & \text{for\ rate-dependent\ power\ model} \end{array},\right. \end{aligned}  $$

where *p*_SP_ is a constant coefficient with unit W/(Gbits/s).

#### Power dissipated on PAs

The amount of power consumed by the PAs strongly depends on the power amplifier’s efficiency. Conventionally, the efficiency of a PA is assumed to be a constant over operating range [5, 8, 9, 16, 17]. This assumption leads to the model

8 $$ P_{\text{PA},b}(\mathbf{v}) \triangleq \frac{1}{\epsilon}\sum_{u\in{\mathcal {U}}_{b}}\|\mathbf{v}_{b_{u}}\|_{2}^{2},  $$

where *P*_PA,*b*_ denotes the PAs’ dissipated power at BS *b*, and *ε*∈(0,1) is a constant standing for the PA efficiency. However, in practice, PA efficiency is highly dependent on the output power region and the employed PA type. To account this, the nonlinear power consumption models of PAs have been introduced [21–24] in which the PA efficiency of RF chain *m* at BS *b* is expressed as

9 $$ \mathrm{{\epsilon}}_{b,m}(\{\mathbf{v}_{b_{u}}\}_{u}) \triangleq \tilde{\epsilon}\sqrt{\sum_{u\in{\mathcal {U}}_{b}}|[\mathbf{v}_{b_{u}}]_{m}|^{2}},  $$

where $\tilde {\epsilon }=\epsilon _{\max }/\sqrt {P_{b}^{m}}$, and *ε*_max_∈(0,1) is the maximum PA’s efficiency. We note that $P_{b}^{m}$ and *ε*_max_ depend on the employed PA techniques. For notational simplicity, we assume that $\tilde {\epsilon }$ is the same for all *b* and *m*. From (9), the total power consumption on the PAs at BS *b* can be written as

10 $$ P_{\text{PA},b}(\mathbf{v}) \!\triangleq\! \sum_{m=1}^{M}\frac{\sum_{u\in{\mathcal {U}}_{b}}|[\mathbf{v}_{b_{u}}]_{m}|^{2}}{\mathrm{{\epsilon}}_{b,m}(\{\mathbf{v}_{b_{u}}\}_{u})}\,=\,\frac{1}{\tilde{\epsilon}}\sum_{m=1}^{M}\sqrt{\sum_{u\in{\mathcal {U}}_{b}}|[\mathbf{v}_{b_{u}}]_{m}|^{2}}.  $$

#### General power consumption models

Based on the above discussion, the total power consumption model in cell *b* can be collectively written as

11 $$ P_{\text{BS},b}(\mathbf{v}) \triangleq P_{\text{cir},b}+P_{\text{SP},b}(r_{b}(\mathbf{v}))+P_{\text{PA},b}(\mathbf{v}).  $$

Hence, the total network power consumption is

12 $$ P_{\text{total}}(\mathbf{v}) \triangleq \sum_{b\in{\mathcal{B}}}\left(P_{\text{cir},b}+P_{\text{SP},b}(r_{b}(\mathbf{v}))+P_{\text{PA},b}(\mathbf{v})\right).  $$

On the other hand, the power for the data transmission to a user is a favorable measure in some user-centric applications. Let

$$\begin{aligned} P_{\text{SP},b}(r_{b_{u}}(\mathbf{v}))\triangleq \left\{\begin{array}{ll} P_{\text{SP},b}(r_{b}(\mathbf{v}))/U & \text{for\ fixed\ power\ model}\\ {p_{\text{SP}}r_{b_{u}}(\mathbf{v})} & \text{for\ rate-dependent\ power\ model} \end{array}\right. \end{aligned} $$

denote the signal processing power corresponding to user *b*_*u*_. Then, the amount of consumed power corresponding to the data transmission to user *b*_*u*_ can be written as [17]

13 $$\begin{array}{*{20}l} P_{\text{Us},b_{u}}(\mathbf{v}) \triangleq& \sum_{m=1}^{M} \frac{|[\mathbf{v}_{b_{u}}]_{m}|^{2}}{\tilde{\epsilon}\sqrt{\sum_{u\in{\mathcal {U}}_{b}}|[\mathbf{v}_{b_{u}}]_{m}|^{2}}}\\ &+P_{\text{SP},b}(r_{b_{u}}(\mathbf{v}))+\frac{P_{\text{sta}}+{MP}_{\text{dyn}}}{U}+P_{\text{Us}} \end{array} $$

in which all users in a cell are assumed to be evenly responsible for the operating power of their serving BS.

### Energy-efficiency metrics

**Fig. 3:**
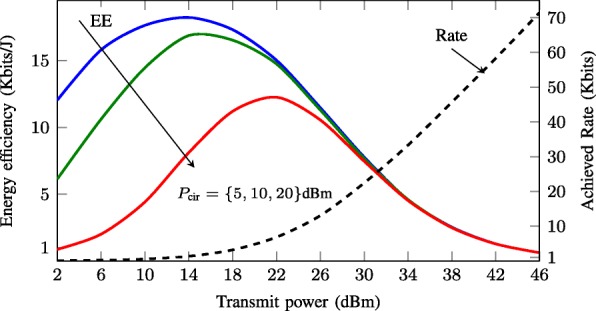
Energy efficiency (solid curves) and the achieved user rate (dashed curve) versus the transmit power for single-cell single-user MISO downlink. The simulation parameters are given in Table 1

The EE measures the number of bits reliably transmitted by a unit energy. In other words, it can be defined as the ratio of the achievable data rate to the total power consumption. The ratio quantifies the trade-off between the network throughput and the power consumption. This is illustrated via a simple single-cell single-user MISO downlink example in Fig. 3. The energy efficiency and the achieved rate are plotted versus the transmit power. We observe that, for all cases of the operating circuit power, when the transmit power increases, the EE first increases, reaching a maximum, and then decreases. In other words, when the circuit power plays a non-negligible role and the rate is penalized by the overall power consumption, the optimum performance is not achieved by using all available power budget. This observation gives rise to the systematic development of the optimization algorithms as detailed below, where four different widely considered EE metrics are introduced and discussed.

**Table 1:** Simulation parameters

Parameters	Value
Path loss and shadowing	$38\log _{10}\left (d\mathrm {[m]}\right)+34.5+\mathcal {N}(0,8)$
Inter-BS distance	*D*=1 km
Static power consumption *P*_sta_	33 dBm
Static power consumption *P*_dyn_	30 dBm
Power amplifier efficiency *ε*	0.35
Number of BSs *B*	3
Number of users per cell *U*_*b*_	2
Number of Tx antennas *M*	4
Signal bandwidth *W*	10 kHz
Power spectral density of noise	− 174 dBm/Hz

#### Network energy efficiency

The NEE metric quantifies the EE performance of the entire network [5, 25]. It is defined as

14 $$ \text{NEE}(\mathbf{v})\triangleq\frac{R(\mathbf{v})}{P_{\text{total}}(\mathbf{v})}  $$

We remark that in scenarios where cellular BSs with different features and specifications co-exist, e.g., heterogeneous network, NEE might lack relevance, since neither EE requirement for each cell/user nor the fairness among all parties of the network can be guaranteed.

#### Sum weighted energy efficiency

The SWEE metric can satisfy the specific demand on EE of each network node. For the considered system model, SWEE can be expressed as

15 $$ \begin{aligned} \text{SWEE}(\mathbf{v})\triangleq \left\{\begin{array}{ll} {\sum\limits_{b\in{\mathcal {B}}}\omega_{b}\frac{\sum_{u\in{\mathcal {U}}_{b}}r_{b_{u}}(\mathbf{v})}{P_{\mathrm{BS,}b}(\mathbf{v})}} & \textrm{for\ the\ BS-centric\ network}\\ {\sum\limits_{b\in{\mathcal {B}}}\sum\limits_{u\in{\mathcal {U}}_{b}}\omega_{b_{u}}\frac{r_{b_{u}}(\mathbf{v})}{P_{\text{Us},b_{u}}(\mathbf{v})}} & \textrm{for\ the\ user-centric\ network} \end{array}\right. \end{aligned}  $$

where *ω*_*b*_∈(0,1] and $\omega _{b_{u}}\in (0,1]$ are parameters representing the priority for cell *b* and user *b*_*u*_, respectively.

#### Weighted product EE

The WPEE metric also takes into account the individual demand on EE of each node which is defined as [5, 26]

16 $$ \begin{aligned} \text{WPEE}(\mathbf{v})\triangleq \left\{\begin{array}{ll} {\prod\limits_{b\in{\mathcal {B}}}\left(\frac{\sum_{u\in{\mathcal {U}}_{b}}r_{b_{u}}(\mathbf{v})}{P_{\mathrm{BS,}b}(\mathbf{v})}\right)^{\omega_{b}}} & \textrm{for\ the\ BS-centric\ network}\\ {\prod\limits_{b\in{\mathcal {B}}}\prod\limits_{u\in{\mathcal{U}}_{b}}\left(\frac{r_{b_{u}}(\mathbf{v})}{P_{\text{Us},b_{u}}(\mathbf{v})}\right)^{\omega_{b_{u}}}} & \mathrm{for\ the\ user-centric\ network} \end{array}\right. \end{aligned}  $$

It is worth noting that although the WPEE metric does not give the same EE-unit (bits/J) as such, it has been used in the literature to achieve fairness in EE. Specifically, it is not difficult to see that none of the BSs experiences EE close to zero when WPEE is considered.

#### Max-min fairness energy efficiency

The max-min fairness EE metric provides the best fairness for the considered nodes compared to others. This metric is preferable to the scenarios where EE is critical for each cell, e.g., in cellular networks where BSs are not connected to a fixed electricity grid. The definition of the metric is given as [10]

17 $$ \begin{aligned} \text{minEE}(\mathbf{v})\triangleq \left\{\begin{array}{ll} {\min\limits_{b\in{\mathcal{B}}}\frac{\sum_{u\in{\mathcal {U}}_{b}}r_{b_{u}}(\mathbf{v})}{P_{\mathrm{BS,}b}(\mathbf{v})}} & \textrm{for\ the\ BS-centric\ network}\\ {\min\limits_{\substack{b\in{\mathcal{B}}\\ u\in{\mathcal {U}}_{b} } }\frac{r_{b_{u}}(\mathbf{v})}{P_{\text{Us},b_{u}}(\mathbf{v})}} & \mathrm{for\ the\ user-centric\ network} \end{array}\right. \end{aligned}  $$

### Energy efficiency optimization problems

From the above discussions, the problems of beamforming design for EE maximization can be generally written as

18 $$ \underset{\mathbf{v}}{\text{maximize}}\ \ f_{\text{EE}}(\mathbf{v})\quad\text{subject to}\ \{(4),(5)\}  $$

where the objective function *f*_EE_(**v**) represents one of the NEE(**v**), SWEE(**v**), WPEE(**v**), and minEE(**v**).

In general, (18) is a nontractable fractional program.^3^ In the next section, we briefly review the conventional approaches, which suboptimally solve the EEmax problems, and then provide the recently proposed SCA framework which improves efficiently the solution quality.

## Centralized methods for energy-efficient transmissions: review and extension

### Conventional fractional programming approaches

Most of the existing solutions for the EEmax problems are based on conventional fractional programming methods, i.e., parameterized approaches [5, 8, 9] or the parameter-free approach based on the Charnes-Cooper transformation. We briefly sketch the idea of these approaches for solving fractional programs below.

In general, a fractional program is expressed as

19 $$\begin{array}{*{20}l} \underset{{\mathbf{x}}\in{\mathcal{S}}}{\text{maximize}}\ & \sum_{i=1}^{L}\frac{f_{i}(\mathbf{{x})}}{g_{i}(\mathbf{{x})}} \end{array} $$

where *L*≥1, ${\mathcal {S}}$, *f*_*i*_(**x)** and *g*_*i*_(**x)** are a convex set, concave and convex functions respect to variable vector ${\mathbf {x}\in \mathbb {C}^{N}}$, respectively.

#### Single-ratio fractional programs

When *L*=1, the problem can be transformed into a parameterized form. That is, one can consider the following problem with parameter *ω*: $H(\omega)={\max _{\mathbf {x}\in {\mathcal {S}}}}\{f_{1}(\mathbf {x})-\omega g_{1}(\mathbf {x})\}$. Due to the fact that *H*(*ω*) is continuous and strictly monotonically decreasing [27], *H*(*ω*)=0 has a unique solution *ω*^∗^. The optimal solution to the original fractional program is $\mathbf {x}^{\ast }={\arg \max _{\mathbf {x}\in {\mathcal {S}}}}\{f_{1}(\mathbf {x})-\omega ^{\ast }g_{1}(\mathbf {x})\}$. Thereby, the problem can be solved by finding *ω* such that *H*(*ω*)=0. A parametric approach exploits the Newton method to find root of *H*(*ω*) (often called as the Dinkelbach method or the Newton-Rhapson method). The method first initializes $\omega ^{(0)}=\frac {f_{1}\left (\mathbf {x}^{(0)}\right)}{g_{1}\left (\mathbf {x}^{(0)}\right)}$. Subsequently, the problem *H*(*ω*^(0)^) is solved, the solution of which is then used to update $\omega ^{(n)}=\frac {f_{1}\left (\mathbf {x}^{(n-1)}\right)}{g_{1}\left (\mathbf {x}^{(n-1)}\right)}$, and this procedure is repeated until convergence. Besides the well-known Dinkelbach method, the problem can be also solved as a single convex program using the Charnes-Cooper transformation [28].

#### Multi-ratio fractional programs

When *L*>1, (19) is a sum-of-ratios fractional program. A conventional heuristic strategy for solving this type of problems with concave-convex ratios is to transform it to a parameterized form with some fixed parameters and then search the optimal parameters by solving a series of convex subproblems [9, 29, 30]. Specifically, the solutions for (19) can be obtained by solving as a series of subproblems $H(\boldsymbol {\alpha },\boldsymbol {\beta })={\max _{\mathbf {x}\in {\mathcal {S}}}}\sum _{i=1}^{L}\alpha _{i}(f_{i}(\mathbf {x})-\beta _{i}g_{i}(\mathbf {x}))$, where {*α*_*i*_}_*i*_ and {*β*_*i*_}_*i*_ are parameters. Similarly to the single-ratio case, {*α*_*i*_}_*i*_ and {*β*_*i*_}_*i*_ are first fixed and the subproblem is solved for given parameters. Then, {*α*_*i*_}_*i*_ and {*β*_*i*_}_*i*_ are updated according to a damped Newton method.

Nevertheless, the advantages of the parametric approaches are hardly recognized when they are applied to wireless communications problems because *f*_*i*_(**x**) and *g*_*i*_(**x**) are often nonconvex. Implicitly, the parametric subproblem is nonconvex, and its optimal solutions are difficult to find. To cope with this, the SCA or alternating optimization method based on iterative weighted minimum mean square error (WMMSE) approach is often combined with the parametric method leading to multi-level iterative algorithms. Thus, these algorithms need a very high number of iterations to converge. Moreover, likely local optimality for each parametric problem is achieved which means that parametric approaches may not always guarantee the convergence.

To avoid the multi-level iterative procedure, we present below the framework developed recently based on the SCA method. The algorithms derived from the approach are provably and fast convergent; thus, they overcome the issues raised by the earlier solutions.

### SCA principle

We first briefly review the SCA principles before presenting their applications to the EEmax problems. The central idea of the SCA method is to iteratively approximate the nonconvex constraints of an optimization problem by proper convex ones [31]. In particular, let us consider a general optimization program given by

20 $$ \underset{\mathbf{x}\in{\mathcal{S}}}{\text{minimize}}\enskip f(\mathbf{x})\quad\text{subject to}\quad\{g_{i}(\mathbf{x})\leq0,\ i=1,\ldots,L\}  $$

where *f*(**x**) is convex and {*g*_*i*_(**x**)}_*i*_ are nonconvex functions in a convex set ${\mathcal {S}}$ w.r.t variable vector **x**. At iteration *n*, given a feasible point **x**^(*n*)^, function *g*_*i*_(**x**) is approximated by its convex approximation function $\hat {g}_{i}(\mathbf {x},\mathbf {x}^{(n)})$ for all *i* such that

- **(a)** $g_{i}(\mathbf {x})\leq \hat {g}_{i}\left (\mathbf {x},\mathbf {x}^{(n)}\right)$
- **(b)** $g_{i}(\mathbf {x}^{(n)})=\hat {g}_{i}\left (\mathbf {x}^{(n)},\mathbf {x}^{(n)}\right)$
- **(c)** $\nabla _{\mathbf {x}}g_{i}(\mathbf {x}^{(n)})=\nabla _{\mathbf {x}}\hat {g}_{i}\left (\mathbf {x}^{(n)},\mathbf {x}^{(n)}\right)$

for all $\mathbf {x}\in \tilde {{\mathcal {S}}}\triangleq \{\mathbf {x}\in {\mathcal {S}}|g_{i}(\mathbf {x})\leq 0,\ i=1,\ldots,L\}$. Properties (a) and (b) are to guarantee the monotonic (objective) convergence behavior for the SCA algorithm; properties (b) and (c) guarantee that the Karush-Kuhn-Tucker (KKT) optimality conditions are satisfied by convergent points [31]. By the replacement, we arrive at the following convex subproblem

21 $$ \underset{\mathbf{x}\in{\mathcal{S}}}{\text{minimize}}\enskip f(\mathbf{x})\quad\text{subject to}\!\quad\{\hat{g}_{i}(\mathbf{x},\mathbf{x}^{(n)})\leq0,\ i=1,\ldots,L\}.  $$

The optimal solution **x**^∗^ of (21) belongs to the set $\tilde {{\mathcal {S}}}$ due to (a) and (b). Thus, **x**^∗^ is used as the feasible point for the next iteration, i.e., **x**^(*n*+1)^=**x**^∗^. The process is iteratively carried out until convergence is established. The SCA procedure solving (20) is outlined in Algorithm 1. We note that *f*(**x**^∗^)≤*f*(**x**^(*n*)^) for all *n*, i.e. sequence {*f*(**x**^(*n*)^)}_*n*_ decreases monotonically. Thus, {*f*(**x**^(*n*)^)}_*n*_ converges if it is bounded below by a finite value in the set $\tilde {{\mathcal {S}}}$. The following remark shows a well-known method for arriving $\hat {g}_{i}(\mathbf {x},\mathbf {x}^{(n)})$, which is widely used in this paper.


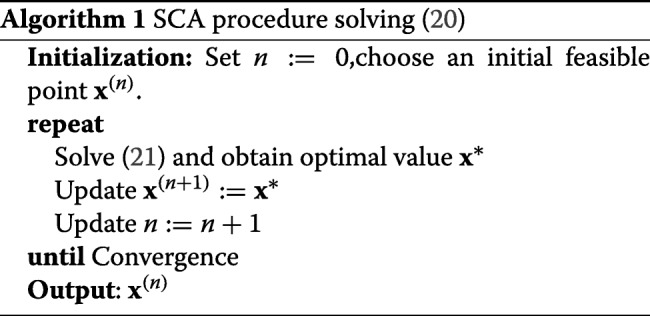


#### **Remarks 1**

Let *g*(**x**)be a concave function w.r.t **x**, then its convex upper bound satisfying (a)–(c) can be achieved by the mean of the first-order Taylor approximation as

22 $$\begin{array}{*{20}l} g(\mathbf{x}) & \leq\underset{\triangleq\hat{g}(\mathbf{x};\mathbf{x}^{(n)})}{\underbrace{g(\mathbf{x}^{(n)})+\left\langle \nabla_{\mathbf{x}}g(\mathbf{x}^{(n)}),\mathbf{x}-\mathbf{x}^{(n)}\right\rangle }}. \end{array} $$

#### **Example 1**

Consider the quadratic-over-linear function $g(x,y)=\frac {-x^{2}}{y}$, *y*>0, which is concave w.r.t the involved variables. From (22), a convex upper bound of *g*(*x*,*y*) at (*x*^(*n*)^,*y*^(*n*)^), *y*^(*n*)^>0, is written as

$$\begin{aligned} \hat{g}(x,y;x^{(n)},y^{(n)}) & =-\left(\!\!\frac{\left(x^{(n)}\right)^{2}}{y^{(n)}}\,+\,\frac{2x^{(n)}}{y^{(n)}}\left(x-x^{(n)}\right)\, -\,\frac{\left(x^{(n)}\right)^{2}}{\left(y^{(n)}\right)^{2}}\left(y-y^{(n)}\right)\!\right)\\ & =-\frac{2x^{(n)}}{y^{(n)}}x+\frac{(x^{(n)})^{2}}{(y^{(n)})^{2}}y. \end{aligned} $$

It can be easily justified that $\hat {g}\left (x,y;x^{(n)},y^{(n)}\right)$ satisfies properties (a)–(c) for all (*x*,*y*>0).

### SCA-based solutions for EEmax problems

In this section, we present how to adopt the procedure discussed in Section 3.2 to the EEmax problems posted in Section 2.4. It is worth mentioning that directly applying the SCA method to these problems seems challenging, because deriving convex approximations for nonconvex parts in the problems that satisfy conditions (a)–(c) is very difficult. Thus, the necessary step is to transform the EEmax problems into more tractable representations, which preserve the optimality of the original one as well as are amenable to the SCA method.

#### Network EEmax problem

We first provide the SCA solutions for the problem with network EE metric which contains single-ratio fractional objective. Replacing *f*_EE_(**v**) in (18) by NEE(**v**), we get the following problem

23 $$ \underset{\mathbf{v}}{\text{maximize}}\enskip\frac{R(\mathbf{v})}{P_{\text{total}}(\mathbf{v})}\quad\text{subject to}\enskip\left\{(4),(5)\right\}.  $$

For translating (23) to a more tractable form, we exploit the epigraph transformation [32]. Let us introduce new slack variables *η*, *z*, *t*, and $\{g_{b_{u}}\}_{b_{u}}$ and rewrite (23) as

24a $$\begin{array}{*{20}l} \underset{\mathbf{v},\eta,z,t,\{g_{b_{u}}\}}{\text{maximize}} & \quad\eta \end{array} $$

24b $$\begin{array}{*{20}l} \mathrm{subject\ to} & \quad\eta\leq\frac{z^{2}}{t}  \end{array} $$

24c $$\begin{array}{*{20}l} & \quad t\geq P_{\text{total}}(\mathbf{v}) \end{array} $$

24d $$\begin{array}{*{20}l} & \quad z^{2}\leq\sum_{b\in{\mathcal{B}},u\in{\mathcal{U}}_{b}}\log(1+g_{b_{u}}) \end{array} $$

24e $$\begin{array}{*{20}l} & \quad\frac{|\mathbf{h}_{b,b_{u}}\mathbf{v}_{b_{u}}|^{2}}{g_{b_{u}}}\!\geq\! G_{b_{u}}(\mathbf{v})\,+\,\sigma_{b_{u}}^{2},\ \forall b\in{\mathcal{B}},u\in{\mathcal{U}}_{b} \end{array} $$

24f $$\begin{array}{*{20}l} & \quad(4),(5). \end{array} $$

The relationship between (23) and (24) is stated in the following lemma.

##### **Lemma 1**

Problems (23) and (24) are equivalent at optimality.

The proof of the lemma is given in the Appendix. Let us now apply the SCA method to solve (24). First, we observe that constraints (24b) and (24e) are nonconvex while the others are convex. Second, the nonconvex parts in (24b) and (24e) are in the form of quadratic-over-affine function mentioned in Example 1. Therefore, the valid convex approximations for (24b) and (24e) are given as

25 $$\begin{array}{*{20}l} \frac{2z^{(n)}}{t^{(n)}}z-\frac{(z^{(n)})^{2}}{(t^{(n)})^{2}}t & \geq\eta \end{array} $$

26 $$\begin{array}{*{20}l} \frac{2\mathfrak{R}(\tilde{\mathbf{h}}_{b,b_{u}}^{(n)}\mathbf{v}_{b_{u}})}{g_{b_{u}}^{(n)}}-\frac{|\mathbf{h}_{b,b_{u}}\mathbf{v}_{b_{u}}^{(n)}|{}^{2}g_{b_{u}}}{(g_{b_{u}}^{(n)})^{2}} & \geq G_{b_{u}}(\mathbf{v})+\sigma_{b_{u}}^{2} \end{array} $$

respectively, where $\tilde {\mathbf {h}}_{b,b_{u}}^{(n)}\!\triangleq \!\left (\!\mathbf {v}_{b_{u}}^{(n)}\! \right)^{\!H}\!\mathbf {h}_{b,b_{u}}^{H}\mathbf {h}_{b,b_{u}}$ and $\left ({\vphantom {\left \{\! g_{b_{u}}^{(n)} \!\right \}}}\mathbf {v}^{(n)},z^{(n)}, {t}^{(n)},\left \{\! g_{b_{u}}^{(n)} \!\right \} \!\right)$ is some feasible point of (24). As a result, we arrive at the approximate convex program at iteration *n* as

27 $$\begin{array}{*{20}l} \underset{\mathbf{v},\eta,z,t,\{g_{b_{u}}\}}{\text{maximize}} & \enskip\eta\quad\text{subject to}\ \{(4),(5),(24\text{c}),(24\text{d}),(25),(26)\}. \end{array} $$

For the rate-dependent signal processing model, due to the following relation $\arg \max _{\mathbf {v}}\frac {R(\mathbf {v})}{P_{\text {total}}(\mathbf {v})}=\arg \min _{\mathbf {v}}\frac {P_{\text {total}}(\mathbf {v})}{R(\mathbf {v})}=\arg \min _{\mathbf {v}}\frac {\sum _{b\in {\mathcal {B}}}\left (P_{\text {cir},b}+P_{\text {PA},b}(\mathbf {v})\right)}{R(\mathbf {v})}+p_{\text {SP}}=\arg \max _{\mathbf {v}}\frac {R(\mathbf {v})}{\sum _{b\in {\mathcal {B}}}\left (P_{\text {cir},b}+P_{\text {PA},b}(\mathbf {v})\right)}$, we can ignore the term of rate-dependent power in the optimization process without loss of optimality. Consequently, the denominator of the objective becomes a convex function w.r.t. **v**, and thus, the solutions can be obtained following the above discussion.

#### Sum weighted EEmax problem

We focus on the problem of SWEE maximization from the perspective of the BSs. The SWEE maximization problem from the user perspective is treated similarly. Replacing *f*_EE_(**v**) in (18) by SWEE(**v**), we arrive at the problem

28 $$ \underset{\mathbf{v}}{\text{maximize}}\enskip\sum_{b=1}^{B}\omega_{b}\frac{\sum_{u\in{\mathcal {U}}_{b}}r_{b_{u}}(\mathbf{v})}{P_{\text{BS,}b}(\mathbf{v})}\quad\text{subject to}\enskip\left\{(4),(5)\right\}.  $$

As the first step, we introduce new variables {*η*_*b*_}_*b*_,{*z*_*b*_}_*b*_,{*t*_*b*_}_*b*_, and $\{g_{b_{u}}\}_{b_{u}}$ and write (28) in equivalent form as

29a $$\begin{array}{*{20}l} \underset{\mathbf{v},\{\eta_{b}\},\{z_{b}\},\{t_{b}\},\{g_{b_{u}}\}}{\text{maximize}} & \quad\sum_{b\in{\mathcal{B}}}\omega_{b}\eta_{b} \end{array} $$

29b $$\begin{array}{*{20}l} \text{subject to} & \quad\eta_{b}\leq\frac{z_{b}^{2}}{t_{b}},\ \forall b\in{\mathcal{B}} \end{array} $$

29c $$\begin{array}{*{20}l} & \quad t_{b}\geq P_{\text{BS},b}(\mathbf{v}),\ \forall b\in{\mathcal{B}} \end{array} $$

29d $$\begin{array}{*{20}l} & \quad z_{b}^{2}\leq\sum_{u\in{\mathcal{U}}_{b}}\log(1+g_{b_{u}}),\ \forall b\in{\mathcal{B}} \end{array} $$

29e $$\begin{array}{*{20}l} & \quad\frac{|\mathbf{h}_{b,b_{u}}\mathbf{v}_{b_{u}}|^{2}}{g_{b_{u}}}\!\geq\! G_{b_{u}}(\mathbf{v})\,+\,\sigma_{b_{u}}^{2},\ \forall b\!\in\!{\mathcal{B}},u\!\in\!{\mathcal{U}}_{b} \end{array} $$

29f $$\begin{array}{*{20}l} & \quad(4),(5). \end{array} $$

The equivalence between (28) and (29) can be easily justified following the procedure in the proof for Lemma 1. The nonconvex parts of problem (29) lie in (29b) and (29e) which can be approximated in convex forms as

30 $$\begin{array}{*{20}l} \frac{2z_{b}^{(n)}}{t_{b}^{(n)}}z_{b}-\frac{\left(z_{b}^{(n)}\right)^{2}}{\left(t_{b}^{(n)}\right)^{2}}t_{b} & \geq\eta_{b},\ \forall b\in{\mathcal{B}} \end{array} $$

31 $$ \begin{aligned} \frac{2\mathfrak{R}\left(\tilde{\mathbf{h}}_{b,b_{u}}^{(n)}\mathbf{v}_{b_{u}}\right)}{q_{b_{u}}^{(n)}}-\frac{|\mathbf{h}_{b,b_{u}}\mathbf{v}_{b_{u}}^{(n)}|{}^{2}q_{b_{u}}}{\left(q_{b_{u}}^{(n)}\right)^{2}} & \geq G_{b_{u}}(\mathbf{v})\,+\,\sigma_{b_{u}}^{2},\ \forall b\in{\mathcal{B}},u\in{\mathcal{U}}_{b} \end{aligned}  $$

respectively. Then, the subproblem solved in iteration *n* is

32 $$ \begin{aligned} &\underset{\mathbf{v},\{\eta_{b}\},\{z_{b}\},\{t_{b}\},\left\{g_{b_{u}}\right\}}{\text{maximize}} \sum_{b\in{\mathcal{B}}}\omega_{b}\eta_{b}\\ &\qquad\text{subject to}\ \{({4}),(5),(\text{29c}),(\text{29d}),(30),(31)\}. \end{aligned}  $$

For the rate-dependent signal processing model, we replace constraints in (29d) and (29c) by

$$\begin{array}{rl} t_{b}+z_{b}^{2}p_{\text{SP}} & \leq\frac{z_{b}^{2}}{\eta_{b}},\ \forall b\in{\mathcal{B}}\\ t_{b} & \geq P_{\text{cir},b}+P_{\text{PA},b}(\mathbf{v}),\ \forall b\in{\mathcal{B}} \end{array}. $$

The same transformation can be applied also to the following problems.

#### Weighted product EEmax problem

WPEE metric has been considered in power control problems so far [5, 26]. However, to the best of our knowledge, beamforming designs for WPEE maximization have not been yet investigated. We show below that the proposed framework can be straightforwardly applied to the problem with such metric. The problem of beamforming designs for WPEE maximization reads

33 $$ \underset{\mathbf{v}}{\text{maximize}}\enskip\prod_{b\in{\mathcal {B}}}\left(\frac{\sum_{u\in{\mathcal {U}}_{b}}r_{b_{u}}(\mathbf{v})}{P_{\text{BS,}b}(\mathbf{v})}\right)^{\omega_{b}}\quad\!\text{subject to}\enskip\left\{(4),(5)\right\}.  $$

Also, we introduce new slack variables {*η*_*b*_},{*z*_*b*_},{*t*_*b*_}, and $\{g_{b_{u}}\}$ then translate (33) into a more tractable form given as

34 $$ \begin{aligned} &\underset{\mathbf{v},\{\eta_{b}\},\{z_{b}\},\{t_{b}\},\left\{g_{b_{u}}\right\}}{\text{maximize}}\enskip\prod_{b\in{\mathcal {B}}}(\eta_{b})^{\omega_{b}}\\ &\!\!\qquad\quad\text{subject to} \left\{(4),(5),(\text{29b}),(\text{29c}),(\text{29d}),(\text{29e})\right\}. \end{aligned}  $$

Again, we can justify the equivalence between (33) and (34) at the optimum similar to that for Lemma 1. We note that the objective function of (34) is generally neither concave nor convex since the exponents {*ω*_*b*_}_*b*_ are arbitrary positive values. A simple way to overcome the issue is to scale the exponents so that the objective function turns into a concave monomial function which is conic quadratic representable [33]. Particularly, we can always find *α*>1 such that $\tilde {\omega }_{b}=\frac {\omega _{b}}{\alpha }$ for all *b* and $\sum _{b}\tilde {\omega }_{b}\leq 1$. Then, $\prod _{b\in {\mathcal {B}}}(\eta _{b})^{\tilde {\omega }_{b}}$ is concave monomial. We also note that the optimal solution to (34) stays the same under the scale. Now, we are ready to arrive at the convex subproblem solved at iteration *n* of the SCA algorithm given as

35 $$ \begin{aligned} &\underset{\mathbf{v},\{\eta_{b}\},\{z_{b}\},\{t_{b}\},\{g_{b_{u}}\}}{\text{maximize}} \quad\prod_{b\in{\mathcal {B}}}(\eta_{b})^{\tilde{\omega}_{b}}\\ &\qquad\quad\text{subject to}\ \{(4),(5),(\text{29c}),(\text{29d}),(30),(31)\}. \end{aligned}  $$

#### Max-min fairness energy efficiency

The problem of maxminEE is given by

36 $$ \underset{\mathbf{v}}{\text{maximize}}\enskip\underset{b\in{\mathcal{B}}}{\text{min}}\frac{\sum_{u \in {\mathcal{U}}_{b}}r_{b_{u}}(\mathbf{v})}{P_{\text{BS,}b}(\mathbf{v})}\quad\text{subject to}\enskip\left\{(4),(5)\right\}.  $$

We note that the centralized and distributed solutions for (36) have been provided in [10] and [11], respectively. Here, for complete discussion and self containment, we provide the main steps of solving the problem. Specifically, with the newly introduced variables *η*,{*z*_*b*_},{*t*_*b*_}, and $\{g_{b_{u}}\}$, (36) can be equivalently written as

37 $$ \begin{aligned} &\underset{\mathbf{v},\eta,\{z_{b}\},\{t_{b}\},\{g_{b_{u}}\}}{\text{maximize}}\enskip\eta\\ &\quad\!\!\text{subject to}\enskip\left\{\eta\leq\frac{z_{b}^{2}}{t_{b}},\ \!\forall b\in{\mathcal{B}};(4),(5),(\text{29c}),(\text{29d}),(\text{29e})\right\} \end{aligned}  $$

where *η* represents the minimum EE among all BSs. A convex approximation of (37) solved in iteration *n* is

38 $$ \begin{aligned} \underset{\mathbf{v},\eta,\{z_{b}\},\{t_{b}\},\{g_{b_{u}}\}}{\text{maximize}} & \enskip\eta\quad\text{subject to}\ \{(4),(5),(\text{29c}),(\text{29d}),(30),(31)\}.  \end{aligned}  $$

### SOCP formulations of approximate programs

It is clear that the convex approximate problems (27), (32), (35), and (38) are general convex programs due to the logarithmic constraints, i.e., (24d) and (29d), and the nonlinear model of PA’s efficiency in (24c) and (29c). Although off-the-shelf solvers are applicable to solve such programs, the computational complexity to output the solutions is relatively high in general [33]. Interestingly, it turns out that these constraints can be represented by second-order cone (SOC) constraints which can take the advantages of more powerful SOCP solvers to reduce the computational effort. In the rest of this section, we discuss the methods that can invoke the hidden SOC representation of the approximated convex programs.

We first consider constraint (29c) which can be equivalently transformed as

39 $$\begin{array}{*{20}l} (\text{29c})\!\Leftrightarrow\!\left\{\begin{array}{l} \sqrt{\sum_{u\in{\mathcal {U}}_{b}}|[\mathbf{v}_{b_{u}}]_{m}|^{2}}\!\leq\! u_{b,m},\forall b\in{\mathcal{B}},m=1,\ldots,M\\ t_{b}\geq\sum_{m=1}^{M}u_{b,m}+P_{\text{cir},b},\forall b\in{\mathcal{B}} \end{array}\right. \end{array} $$

where {*u*_*b*,*m*_}_*b*,*m*_ are slack variables. We can see that the first type of constraint in the equivalent formulation is SOC while, the second one is linear. Constraint (24c) is treated similarly and skipped for conciseness.

We now focus on constraint (29d) whose equivalent formulation is given as

40 $$\begin{array}{*{20}l} (\text{29d})\Leftrightarrow\left\{\begin{array}{l} z_{b}^{2}\leq\sum_{u\in {\mathcal{U}}_{b}}\beta_{b_{u}},\forall b\in{\mathcal{B}}\\ \log\left(1+g_{b_{u}}\right)\geq\beta_{b_{u}},\forall b_{u} \end{array}\right. \end{array} $$

where $\{\beta _{b_{u}}\}_{b_{u}}$ are slack variables. Remark that (40) is SOC representable ([33], Section 3.3). Because the first type of constraint on the right side is SOC representable, we only have to deal with the second one. From now on, for notational convenience, we consider constraint log(1+*x*)≥*y* where *x* and *y* are positive variables. In the following, we provide four different approaches translating the constraint into SOC representations.

#### Conic approximation of exponential cone

The first approach approximates log(1+*x*)≥*y* by a set of conic constraints based on the result in ([34], Example 4), which has been particularly applied to reduce complexity of solving EE problems in ([7], (31)) and ([10], (13)). The detailed formulation of the conic constraints approximating logarithmic function is omitted here due to the space limitation.

In some settings, using conic approximation of exponential cone could cause a significant increase in per-iteration complexity due to a large number of additional slack variables. This issue is avoided by the approaches presented following.

#### Equivalently SCA-applicable constraint

The second approach equivalently rewrites log(1+*x*)≥*y* as a nonconvex but SCA-applicable constraint. To see this, let us multiply both sides of the constraint by *x*, i.e.,

41 $$\begin{array}{*{20}l} x\log(1+x)\geq xy  \end{array} $$

Since *x* log(1+*x*) is convex, we can apply SCA principles on (41). A lower bound of *x* log(1+*x*) is given as

42 $$ x\log(1+x)\geq d^{(n)}x-c^{(n)}  $$

where $c^{(n)}\triangleq \frac {\left (x^{(n)}\right)^{2}}{x^{(n)}+1}$, $d^{(n)}\triangleq \frac {x^{(n)}}{x^{(n)}+1}+\log \left (1+x^{(n)}\right)$, and *x*^(*n*)^ is some positive value. Then, an approximation of (41) is

43 $$ d^{(n)}x-c^{(n)}\geq xy  $$

which can be represented as SOC constraint, i.e,

44 $$ \|[x+y-d^{(n)}\hspace{1em}2\sqrt{c^{(n)}}]\|_{2}\leq x-y+d^{(n)}.   $$

#### Concave lower bound of the logarithm

We can use the well-known inequality of logarithmic function given as

45 $$ \log(1+z)\geq z(1+z)^{-1}  $$

for all *z*>− 1. By replacing *z* on both sides of (45) by $\frac {x-x^{(n)}}{x^{(n)}+1}$ we arrive at

46 $$ \log(1+x)\geq\log(1+x^{(n)})+(x-x^{(n)})(1+x)^{-1}  $$

for all *x*≥0. Now, we can easily check that (46) satisfies the three conditions (a)–(c). Thus, the valid approximate of log(1+*x*)≥*y* is

47 $$ \log\left(1+x^{(n)}\right)+\left(x-x^{(n)}\right)(1+x)^{-1}\geq y.  $$

Interestingly, (47) contains a hidden SOCP representation given as

48 $$ {}\|2\sqrt{1+x^{(n)}},\ \log(1+x^{(n)})-y-x\|_{2}\leq\log(1+x^{(n)})-y+x+2.  $$

#### Quadratic lower-bound of the logarithm

We can directly approximate (29d) under SCA principles without requiring the transformation step (40). Specifically, we use the following concave quadratic lower-bound derived based on the Lipschitz continuity of the logarithm [25]

49 $$ \log(1+x)\geq\log\left(1+x^{(n)}\right)+\frac{\left(x-x^{(n)}\right)}{1+x^{(n)}}-\frac{C}{2}\left(x-x^{(n)}\right)^{2}.  $$

With *C*≥1, the inequality holds for all *x*≥0 and *x*^(*n*)^≥0. As a result, an approximation of (29d) can be written as

50 $$ {\!~\!}_{b}^{2}+\frac{C}{2}\left(g_{b_{u}}-g_{b_{u}}^{(n)}\right)^{2}\leq\sum_{u\in {\mathcal{U}}_{b}}\log\left(1+g_{b_{u}}^{(n)}\right)+\frac{\left(g_{b_{u}}-g_{b_{u}}^{(n)}\right)}{1+g_{b_{u}}^{(n)}}  $$

which is indeed a rotated SOC constraint. It is worth noting that constant *C* has large impact on the tightness of the approximation (49), and thus, it influences the convergence speed of the iterative algorithm. More specifically, a smaller value of *C* implies a tighter approximation and may increase the convergence speed (the discussion is numerically justified in Fig. 7).

### Distributed implementation

The algorithms in Section 3.3 are designed in a centralized fashion under the assumption that each BS (or a central controller) perfectly knows all the channel state information in the network. From the practical perspective, distributed solutions may be more attractive. Note that the conventional approaches are not suitable for decentralized implementation, since updating the parameterized values requires a central node [35, 36]. In contrast, the SCA-based algorithms can be easily carried out in distributed manner. In fact, distributed implementations of the SCA solutions for EEmax problems have been provided in [25, 37, 38]. We remark that distributed implementation is preferred to SWEE, WPEE, and EE-fairness due to their goal of achieving EE of individual node. In what follows, we present how to solve SWEE maximization problem (28) distributively and note that the procedure can be applied to WPEE and EE-fairness problems with slight modifications.

We first assume that each BS has (perfect) CSIs of the channels from itself to all users in the network, which is referred to as local CSI. This is a basic assumption in the distributed setting which has been adopted in [25, 37, 38]. The main idea of the proposed approach is to solve the convex subproblem (32) distributively using the alternating direction method of multipliers (ADMM) [39]. To do so, the vital step is to recognize the terms which need to be decoupled. From (32), we observe that excluding the inter-cell interference terms $\{G_{b_{u}}(\mathbf {v})\}$, all the other terms are readily local. For clarity, let us rewrite (31) as

51 $$\begin{array}{*{20}l} &\frac{2\mathfrak{R}(\tilde{\mathbf{h}}_{b,b_{u}}^{(n)}\mathbf{v}_{b_{u}})}{q_{b_{u}}^{(n)}}-\frac{|\mathbf{h}_{b,b_{u}}\mathbf{v}_{b_{u}}^{(n)}|{}^{2}q_{b_{u}}}{(q_{b_{u}}^{(n)})^{2}}\\ \geq&\bar{G}_{b_{u}}(\bar{\mathbf{v}}_{b})+\sum_{k\in{\mathcal{B}}\backslash\{b\}}\sum_{i\in{\mathcal{U}}_{k}}|\mathbf{h}_{k,b_{u}}\mathbf{v}_{k_{i}}|^{2},\ \forall b\in{\mathcal{B}},u\in{\mathcal{U}}_{b} \end{array} $$

where $\bar {\mathbf {v}}_{b}\triangleq \left [\mathbf {v}_{b_{1}}^{T},\mathbf {v}_{b_{2}}^{T},...,\mathbf {v}_{b_{U}}^{T}\right ]^{T}$, and $\bar {G}_{b_{u}}(\bar {\mathbf {v}}_{b})\triangleq \sum _{i\in {\mathcal {U}}_{b}\backslash \{{u}\}} |\mathbf {h}_{b,b_{u}}\mathbf {v}_{b_{i}}|^{2}+\sigma _{b_{u}}^{2}$represents the intra-cell interference plus noise. To deal with the inter-cell interference, let us introduce the variables $\{\theta _{k,b_{u}}\}_{k,b_{u}}$. Then, (51) is equivalent to the following set of constraint

52 $$\begin{array}{*{20}l} &\frac{2\mathfrak{R}\left(\tilde{\mathbf{h}}_{b,b_{u}}^{(n)}\mathbf{v}_{b_{u}}\right)}{q_{b_{u}}^{(n)}}-\frac{|\mathbf{h}_{b,b_{u}}\mathbf{v}_{b_{u}}^{(n)}|{}^{2}q_{b_{u}}}{\left(q_{b_{u}}^{(n)}\right)^{2}}\\ \geq&\bar{G}_{b_{u}}(\bar{\mathbf{v}}_{b})+\sum_{k\in{\mathcal{B}}\backslash\{b\}}\theta_{k,b_{u}},\ \forall b\in{\mathcal{B}},u\in{\mathcal{U}}_{b} \end{array} $$

53 $$\begin{array}{@{}rcl@{}} \theta_{k,b_{u}}\geq\sum_{i\in{\mathcal{U}}_{k}}|\mathbf{h}_{k,b_{u}}\mathbf{v}_{k_{i}}|^{2},\ \forall b\in{\mathcal{B}},u\in{\mathcal{U}}_{b},k\in{\mathcal{B}}\backslash\{b\}. \end{array} $$

With the transformation, we turn to handling the term $\sum _{k\in {\mathcal {B}}\backslash \{b\}}\theta _{k,b_{u}}$ in (52) for distributed implementation since the constraints in (53) can be treated locally. To this end, let us introduce new local variables for each interference term $\theta _{b,k_{i}}$ as $\tilde {\theta }_{b,k_{i}}^{b}$ and $\tilde {\theta }_{b,k_{i}}^{k}$optimized at BS *b* and BS *k*, respectively. To make sure that these local variables are equal to each other, we further add an equality constraint

54 $$\begin{array}{*{20}l} \theta_{b,k_{i}}=\tilde{\theta}_{b,k_{i}}^{b}=\tilde{\theta}_{b,k_{i}}^{k},\forall b\in\mathcal{B},k\in\mathcal{B}\setminus\{b\},i\in{\mathcal{U}}_{k}. \end{array} $$

Now, we can write (32) equivalently as

55a $$\begin{array}{*{20}l} &\text{maximize} \quad\sum_{b\in{\mathcal{B}}}\omega_{b}\eta_{b} \end{array} $$

55b $$\begin{array}{*{20}l} &\text{subject to}\ \left(\!\bar{\mathbf{v}}_{b},\{\eta_{b}\},\{z_{b}\},\{t_{b}\},\{g_{b_{u}}\},\{\tilde{\boldsymbol{\theta}}_{b}\}\!\right)\in{\mathcal{S}_{b}},\forall b\in\mathcal{B} \end{array} $$

55c $$\begin{array}{*{20}l} & \quad\boldsymbol{\theta}_{b}=\tilde{\boldsymbol{\theta}}_{b},\forall b\in\mathcal{B} \end{array} $$

where $\boldsymbol {\theta }_{b}\triangleq \{\{\theta _{k,b_{k}}\}_{k\in \mathcal {B}\setminus \{b\},u\in \mathcal {U}_{b}},\{\theta _{b,k_{i}}\}_{k_{i}\in \bar {\mathcal {U}}_{b}}\}$, $\tilde {\boldsymbol {\theta }}_{b}\triangleq \big \{\{\tilde {\theta }_{k,b_{u}}^{b}\}_{k\in \mathcal {B}\setminus \{b\},u\in \mathcal {U}_{b}},\big \{\tilde {\theta }_{b,k_{i}}^{b}\big \}_{k_{i}\in \bar {\mathcal {U}}_{b}}\big \}$, $\bar {\mathcal {U}}_{b}\triangleq \underset {k\in \mathcal {B}\setminus \{b\}}{\cup }{\mathcal {U}}_{k}$, and

56 $$\begin{array}{@{}rcl@{}} \mathcal{S}_{b} & \triangleq & \left\{(\bar{\mathbf{v}}_{b},\{\eta_{b}\},\{z_{b}\},\{t_{b}\},\{g_{b_{u}}\},\{\tilde{\boldsymbol{\theta}}_{b}\})| \right.  \\ & & \quad \frac{2\mathfrak{R}\left(\tilde{\mathbf{h}}_{b,b_{u}}^{(n)}\mathbf{v}_{b_{u}}\right)}{q_{b_{u}}^{(n)}}- \frac{|\mathbf{h}_{b,b_{u}}\mathbf{v}_{b_{u}}^{(n)}|{}^{2}q_{b_{u}}}{\left(q_{b_{u}}^{(n)}\right)^{2}}\geq\bar{G}_{b_{u}}(\bar{\mathbf{v}}_{b})\\ &&+\sum_{k\in{\mathcal{B}}\backslash\{b\}}\theta_{k,b_{u}}^{b},\ \forall u\in{\mathcal{U}}_{b} \\ & & \quad\theta_{b,k_{i}}^{b}\geq\sum_{u\in{\mathcal{U}}_{b}}|\mathbf{h}_{b,k_{i}}\mathbf{v}_{b_{u}}|^{2},\ \forall k_{i}\in{\bar{{\mathcal{U}}}_{b}} \\ & & \quad \left. {\{(4),(5),(\text{29c}),(\text{29d}),(\text{30}),(\text{31})\}}_{b}\right\} \end{array} $$

is the feasible set for the local variables at BS *b*. In (56), the notation {}_*b*_ means to take only the constraints related to BS *b*. Problem (55) is in a form of global consensus problem and can be optimally solved using the standard ADMM procedure. We refer interested readers to [11, 37] for further details.

## Numerical result

We evaluate the performances of the different algorithms presented above. The general (fixed) simulation parameters are taken from Table 1, and the ones which are changed in the simulations are given in the caption of the corresponding figures. A network of *B* base stations is considered, and *U*_*b*_ users are randomly dropped to the coverage area of each base station. The user channels follow the Rayleigh distribution.

### Comparison on the convergence and the performance

In the first set of simulations, we compare the SCA methods against the conventional based on the fractional program (FP) ones in terms of the convergence rate and achieved EE performances. The following beamforming designs are considered in the evaluation:

- NEE-SCA—the SCA procedure for solving (23).
- SWEE-SCA—the SCA procedure for solving (28). We set equal priorities for all the nodes, i.e, *ω*_*b*_=1,∀*b*.
- WPSEE-SCA—the SCA procedure for solving (33). Similar to SWEE-SCA, *ω*_*b*_=1,∀*b*.
- maxminEE-SCA—the SCA procedure for solving (36).
- NEE-FP—the beamforming design based on FP proposed in [16].
- SWEE-FP—the beamforming design based on FP proposed in [9]. This scheme has been studied for a MIMO channel. However, it can be easily simplified for MISO channels by setting the number of receive antenna to one.
- maxmin-FP—the beamforming design based on FP proposed in [17]. Although this approach has been proposed for a multi-cell joint transmission system, we can easily simplify it for the multi-cell coordinated beamforming case by properly rewriting the signal and interference terms [17] Remark 2.

We note that for the WPEE metric, only the performance of the SCA-based method is studied as beamforming designs for this metric based on the FP framework have not yet been proposed to the best of our knowledge. In addition, to reduce the computational burden for the simulations, we terminate the iterative processes of all the considered algorithms either when the increase in the objective between two consecutive iteration is less than 10^−5^ or after 10^6^ iterations. Also, for a fair comparison, we only consider the conventional power model as in [5, 8, 9, 16, 17], i.e., fixed signal processing power and PAs’ efficiency. The results for the general power consumption model are reported in Section 4.6.

### Convergence comparison of the SCA and PF algorithms

**Fig. 4:**
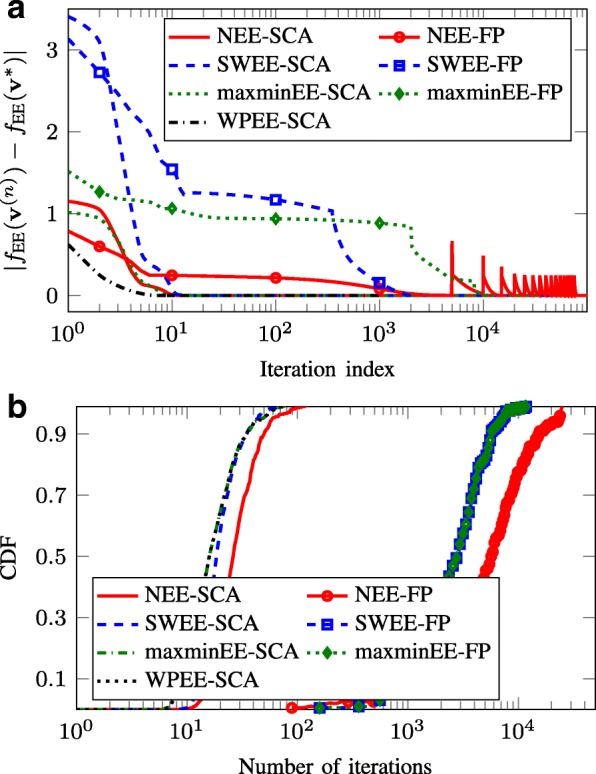
Convergence behavior of different EE schemes with *P*_*b*_=30 dBm. **a** Convergence rate for one channel realization. **b** CDF of the numbers of required iterations over 500 channel realizations

Figure 4 presents the convergence behavior of the considered EE approaches. Particularly, Fig. 4a compares the convergence speed of the SCA frameworks and the FP methods in terms of the number of iterations for one random channel realization by showing the gap between the current objective value at iteration *n* and the achieved objective value after the termination of the algorithms, i.e., |*f*_EE_(**v**^*n*^)−*f*_EE_(**v**^∗^)|. As can be seen, the SCA-based methods have steady monotonic converge properties, e.g., achieve the objective value of the convergence point after ten iterations in the considered setting. For the FP-based approaches, even hundreds or thousands of iterations can be required to reach the convergence, while the monotonic convergence is not always guaranteed, e.g., for the NEE-FP method.

To complete the comparison in terms of convergence speed between the SCA- and FP-based methods, we provide in Fig. 4 the cumulative distribution function (CDF) of the total number of iterations needed for convergence. It is observed that for 90% of channel realizations, the SCA converges after 30 iterations while the FP methods need even thousands of iterations to terminate. This observation again shows the superiority of the SCA algorithms in terms of complexity compared to the conventional approaches.

### EE performance comparison of the SCA and FP algorithms

**Fig. 5:**
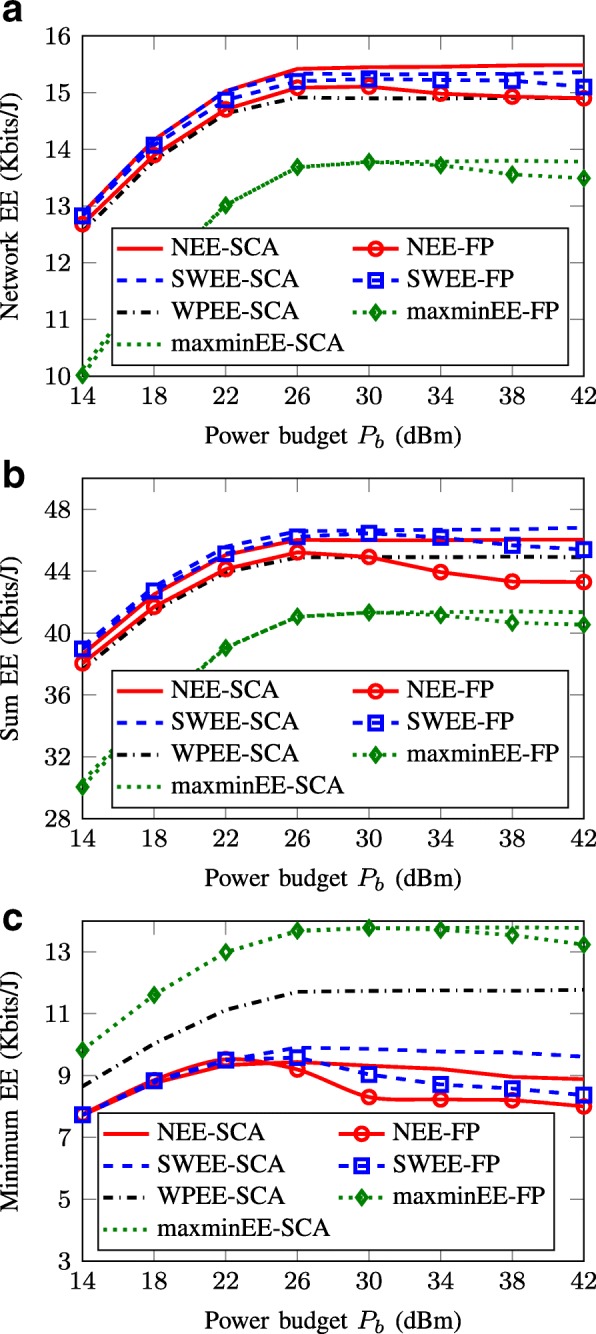
Achieved EE performance versus power budget *P*_*b*_. **a** Network EE. **b** Sum EE. **c** Minimum EE

Figure 5 plots the average performances of the SCA- and FP-based methods in terms of the achieved NEE, the sum EE, and the minimum EE versus the maximum transmit power budget *P*_*b*_. Our first observation is that the SCA method maximizing a specific EE metric achieves approximately the same EE performance compared to the corresponding FP method in small and moderate power regions. This again implies the effectiveness of the SCA framework in solving the EE maximization problems as it can offer similar performance compared to the conventional ones but with much reduced complexity. Another observation is that the achieved EE with all the approaches saturates when the power budget is sufficiently large. This is because in the large power regime, the data rate logarithmically scales with the transmit power while the power consumption increases linearly with the transmit power. Thus, whenever the gain in achieved throughput cannot compensate for the increase of power consumption, the EE methods do not use the excess power to further increase data rate so as to maintain a high value EE. This fact has been discussed in many EE maximization related works [5, 8, 40]. On the other hand, we can see that the EEs achieved by the FP methods slightly downgrade for large value of *P*_*b*_. The reason can be explained as follows. When *P*_*b*_ increases, the feasible set of the EE problems is expanded which results in the increasing number of iterations required for the convergence. However, due to the threshold on the maximum iterations for the iterative process, the FP methods may not reach the suboptimal solutions within 10^6^ iterations. Consequently, they may output poor performances leading to the decrease of average achieved EE value. This observation again points out the drawback of the two-layer iterative procedure in practice. Let us then evaluate the achieved performances of the considered methods with respect to each EE metric. It is obvious that the NEE maximization methods outperform the other schemes in terms of the achieved NEE (in Fig. 5a), while the SWEE methods offer the best sum-EE values (in Fig. 5b). In terms of minimum EE, the maxminEE methods achieve the best performance as they aim at maintaining the balance of EE among all parties (in Fig. 5c). However, the maxminEE approaches suffer a loss in NEE and sum-EE performances. The WPEE metric, as expected, offers a better minimum EE than the SWEE and NEE criteria. We note that individual EE is one of the key features in many network scenarios (e.g., heterogeneous networks), and thus, a per-node EE performance and EE fairness will be the main focus in the next numerical experiment.

### Achieved per-BS EE performance

**Fig. 6:**
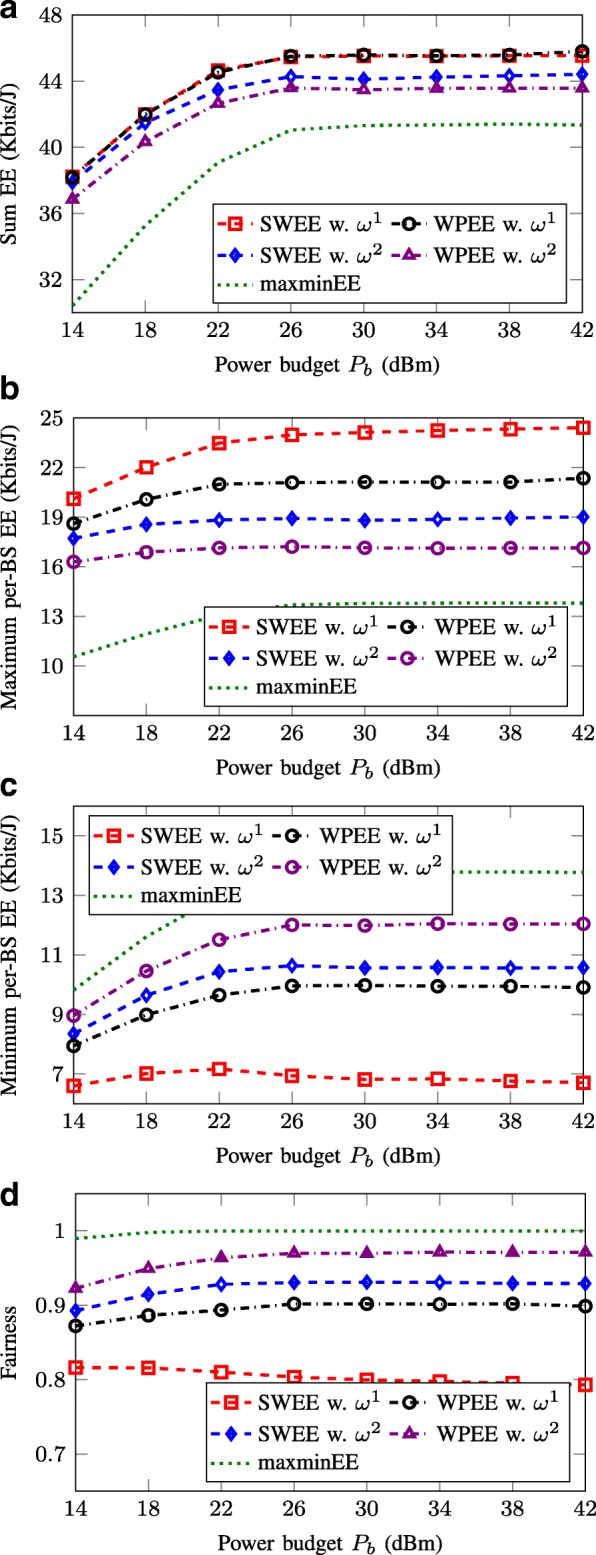
Averaged EE versus transmit power budget *P*_*b*_. For SWEE and WPEE schemes, we use two sets of priority parameters, i.e., ***ω***^1^=[0.7,0.5,0.3] and ***ω***^2^=[0.3,0.5,0.7]. **a** Sum EE. **b** Maximum EE. **c** Minimum EE. **d** Fairness index

In Fig. 6, we compare the achieved fairness when using different fairness-oriented EE metrics, i.e., SWEE, WPEE, and maxminEE, as a function of the maximum transmit power budget *P*_*b*_. Note that the fairness is considered in terms of energy efficiency and not rates. Specifically, we consider two settings of weighting vector, i.e., ***ω***^1^=[0.7,0.5,0.3] and ***ω***^2^=[0.3,0.5,0.7], where each value implies the priority weight for a corresponding BS. In this experiment, users in cell 1 are dropped in the radius of 200 m to its serving BS while for cell 3, users’ locations are near the cell edge. The served users of BS 2 are randomly placed in its coverage region. The figure is plotted to see how the priority parameters alter the per-BS EE behaviors. The average sum-EE, maximum and minimum EE, and EE fairness measure^4^among all the BSs are plotted in Fig. 6a–d, respectively. Similar results as in Fig. 5 can be observed. Particularly, the achieved EE values remain unchanged when *P*_*b*_ is sufficiently large. Moreover, it is seen that the SWEE methods outperform the other schemes in terms of sum-EE and maximum per-BS EE values. This is clear since maximizing the sum of individual EEs is the objective of the SWEE methods. In terms of minimum EE among all nodes, it is obvious that the maxminEE scheme obtains the best performance followed by the WPEE and SWEE criteria (with same assigned priority). Another important observation is that by assigning different priority weights ***ω*** for SWEE and WPEE metrics, we can adjust the achieved EE of each node. It is discovered that with ***ω***^1^, more priority is given to BS 1 leading to an improvement in the sum-EE performance for these two schemes. In addition, since BS 3 is more penalized, the gap between maximum and minimum per-BS EE values is enlarged and, thus, implying high EE unfairness among the BSs. On the contrary, since ***ω***^2^ prioritizes BS 3 and restricts BS 1, it reduces the sum-EE performance of the network but encourages the fairness among all parties. As a conclusion, the EE fairness measure in Fig. 6d shows that the SWEE and WPEE schemes can tune the EE fairness of the system by the priority parameters while the maxminEE can establish the absolute fairness among all the per-BS EEs. Also, with the same weighting vector, the WPEE metric outperforms the SWEE in terms of EE balancing. The WPEE achieves a better trade-off between fairness and EE performance compared to the two other schemes SWEE and maxminEE.

### SCA with different conic approximations

**Fig. 7:**
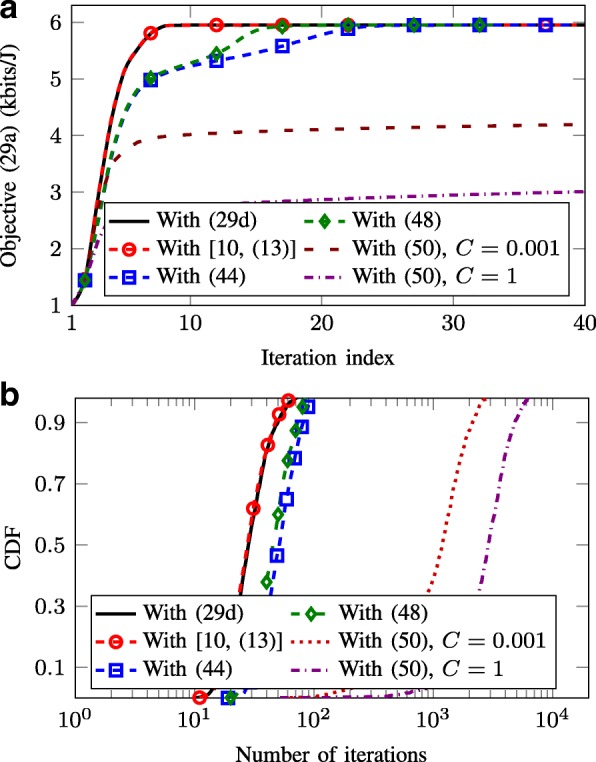
Convergence behavior of the SCA algorithm solving (32) with different conic approximations. We take *P*_*b*_=40 dBm. **a** Convergence rate for one random channel realization. **b** CDF of the number of required iterations over 1000 channel realizations

We now illustrate the performances of the SOCP formulations provided in Section 3.4, by focusing on the SWEE metric. In Fig. 7a, we compare the convergence rate of the objective (29a) when the original logarithmic constraint (29d) and its SOC constraints ([10], (13)), (44), (48), and (50) are used in (32). It is seen that with ([10], (13)), the algorithm converges with the same rate as using (29d) and faster than the other SOC approximations. This is understandable because the set of conic constraints ([10], (13)) is in fact a tight approximation of logarithmic function up to a fixed accuracy level. On the other hand, the other ones are the upper bounds of the logarithmic function which are tight only in the fixed point at each iteration. Between (44) and (48), we can observe that (48) offers a better convergence rate. This may be understood as (48) directly approximates the bound of logarithmic function while (48) is derived from the bound of the equivalent transformation of (29d). For the SOC approximation based on (50), it is seen that the convergence behavior largely depends on *C*. When a large value of *C* is used, the objective slowly converges while the convergence rate significantly improves when using small value of *C*. The performance agrees with the analysis of (50) which argues that the smaller *C* provides a tighter approximation in (50) and, thus, can lead to a faster convergence.

In Fig. 7b, we depict the CDF of the number of iterations required for convergence with different SOCP formulations of (32). As expected, the result is consistent with that observed from Fig. 7a. Specifically, adopting the set of conics constraints ([10], (13)) to approximate the logarithmic function does not require more iterations for convergence compared to using the original constraint (29d). Also, the number of iterations of (32) with (48) is smaller than that of applying (44). In general, we can see that for 90% of the channel realization, the approximation methods ([10], (13)), (44), and (48) can provide a good convergence rate which is smaller than 100 iterations. On the other hand, (50) results in slow convergence speed in the considered setting.

### Achieved performance with general power consumption model

**Fig. 8:**
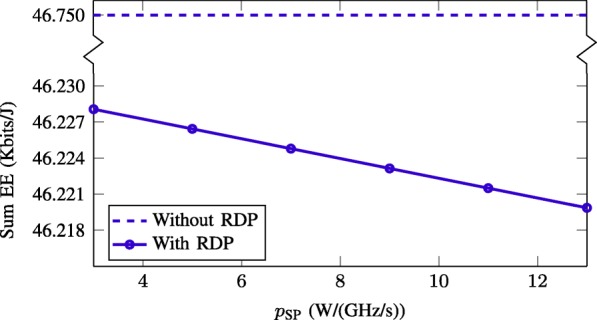
Achieved sum-EE of SWEE scheme with and without considering rate-dependent power consumption. We take *P*_*b*_=40 dBm

In this numerical experiment, we provide insights to the impact of rate-dependent signal processing power and the nonlinear model of PA’s efficiency on the achieved EE performance. Figure 8 compares the achieved sum-EE of the SWEE schemes without and with considering the rate-dependent power (RDP) consumption, which are labeled as “Without RDP” and “With RDP,” respectively. The curve “Without RDP” is obtained by solving problem (29) with *p*_SP_=0. Then, we recalculate the EE values for “With RDP” scheme with the given *p*_SP_ in the horizontal axis. Our observation is that as *p*_SP_ increases, the achieved EE monotonically decreases. This is understandable because, for a fixed achieved data rate, higher *p*_SP_ increases the total power consumption and thus degrades the EE. This result suggests that RDP may be included when optimizing the EE performance of a wireless network.

**Fig. 9:**
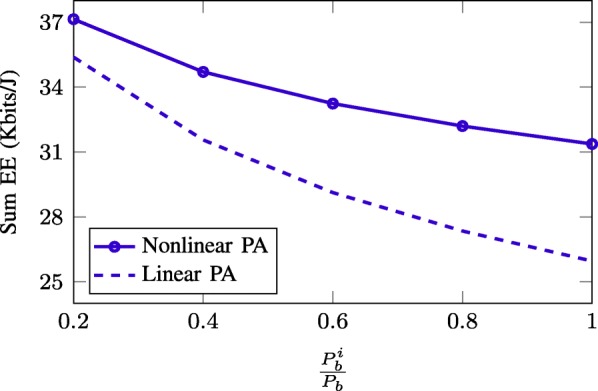
Achieved sum-EE of SWEE scheme versus ratio $\frac {P_{b}^{m}}{P_{b}}$ for two designs considering linear and nonlinear model of PA’s efficiency. We take *P*_*b*_=40 dBm
- “Linear PA”—problem (28) is solved using the PA’s power consumption model (8) with fixed PA’s efficiency *ε*=0.35. The resulting beamforming solution is used to compute the actual EE performance following the PA’s efficiency model (9).

Next, in Fig. 9, we evaluate the impact of nonlinear PA’s efficiency on the sum-EE achieved by the SWEE method. For this purpose, we perform the EE optimization based on the nonlinear PA’s efficiency model (9). As PA’s efficiency does not depend on *P*_*b*_ but $P_{b}^{m}$, we fix *P*_*b*_=40 dBm and plot the achieved sum EE as a function of the ratio $\frac {P_{b}^{m}}{P_{b}}$. The two following schemes are compared:

- “Nonlinear PA”—problem (28) is solved using the nonlinear PA’s efficiency model (9) with *ε*_max_=0.35.

As can be seen, “Linear PA” scheme is inferior to “Nonlinear PA” one which clearly shows that the power modeling has a remarkable influence on the achieved EEs. More specifically, the EE maximization based on the assumption that the PA’s efficiency is the same regardless of the output power potentially degrades the EE performance in practical implementation, where the PA’s efficiency actually depends on the desired output power [21–24]. Another observation is that the achieved EEs of both schemes decrease against the increase of $\frac {P_{b}^{m}}{P_{b}}$. The result can be explained as follows. Recall that the effective PA’s efficiency depends on $P_{b}^{m}$ and the actual transmit power (ATP) on the antenna, that is, with increasing $P_{b}^{m}$, the efficiency slope of that PA is changed so that the efficiency is worse in the lower ATP regime (see (9)). Thus, the decreased PA efficiency simply deteriorates the achieved EE.

### Achieved EE in large-scale network settings

**Fig. 10:**
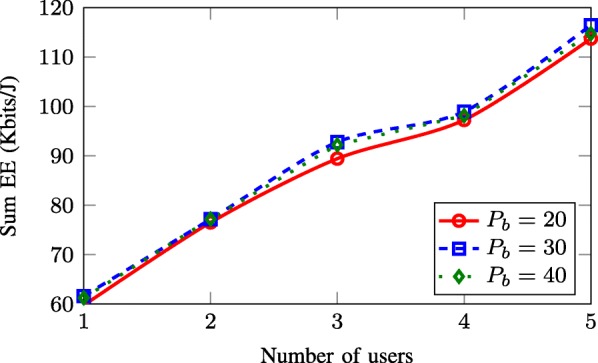
Achieved sum-EE of the SWEE scheme versus the number of users per cell *U*_*b*_ with *B*=7, *M*=4

In the final set of numerical experiments, we illustrate how the achieved EE behaves in a larger network. A seven-cell network model which consists of *B*=7 BSs is considered. For simplicity, we adopt the conventional power consumption model with (8) and *p*_SP_=0 and simulate only the SWEE scheme. Figure 10 shows the achieved EE versus the number of users per cell *U*_*b*_ with different values of transmit power *P*_*b*_ when the number of per-BS antennas is fixed to *M*=4. It is seen that the EE values increase with the increasing number of served users. This is because the sum rate is an increasing function of the number of users and, thus, increased when more users are involved in the transmission. We can also observe that for fixed *U*_*b*_, the EE grows if the power budget is larger. However, when *P*_*b*_ is large enough, further increasing *P*_*b*_ does not bring significant improvement in EE. This result is consistent with that observed in Figs. 5 and 6.

**Fig. 11:**
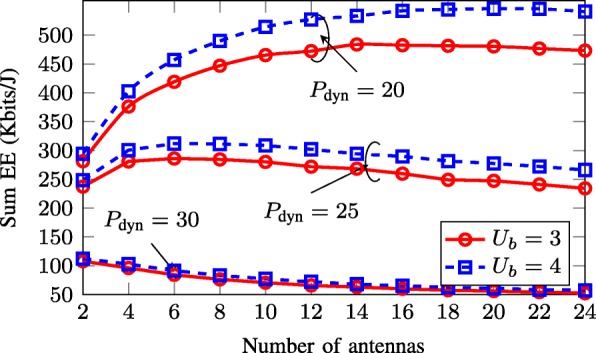
Achieved sum-EE of the SWEE scheme versus the number of antennas per BS with *B*=7. We take *P*_*b*_=40 dBm

In Fig. 11, we show the achieved EE versus the number of per-BS antennas *M* for different values of *P*_dyn_ and *U*_*b*_. As can be seen, for small and moderate values of *P*_dyn_, the achieved EE first increases then decreases as *M* keeps increasing, while for the large value of *P*_dyn_, it monotonically decreases. The reason can be explained as follows. Recall the fact that additional antennas provide more degree of freedom which leads to the improvement in the achievable data rate. However, since the total power consumption linearly scales with the transmit antennas, adding more antennas consumes more circuit power. Thus, as long as the the benefit offered from transmitting with additional antennas is beyond the cost of the power consumption, the achieved EE increases. Otherwise, increasing the number of transmit antennas degrades the achieved EE. Another observation, which agrees with the result in Fig. 10, is that adding more users improves the achieved EE.

## Conclusions

We have provided a summary and performance comparison of various algorithms for the problems of EE optimization in multi-cell multiuser MISO downlink, under four energy efficiency metrics. We have reviewed and presented the SCA framework to provide efficient solutions for the energy efficiency optimization. The algorithms have been numerically evaluated and compared with different fractional programming solutions for the same problems. The SCA-based algorithms have been shown to outperform the existing FP ones in terms of convergence speed. This paper can be viewed as a guideline for the application of the SCA in solving the energy-efficient beamforming designs in particular and the nonconvex problems in wireless communications in general.

The EE optimization will be important for the sustainability of the future digital society. Several important problems still remain. For example, acquiring accurate CSI is challenging in practice, and the transmission designs taking into account the effect of imperfect CSI are an important topic to be explored. The impact of data sharing over (wireless) power and bandwidth-limited backhaul in the CoMP joint processing transmission is also an important topic. The hybrid analog/digital beamforming transceiver architecture-based EE optimization for the evolving millimeter wave wireless communications is an important item for the evolving 5G system design. The cloud radio access network (CRAN) architecture with more processing options either close to the antenna or at computing cloud requires also the EE-based design and analysis. Finally, the power consumption in user devices is much more difficult to model and control than that in the base stations or cloud but constitutes a significant portion of the overall network power consumption.

## Appendix

### Proof of Lemma 1:

For proving the lemma, we show that constraints (24b)–(24e) are active at the optimality by the contradiction. Let $(\mathbf {v}^{\ast },\eta ^{\ast },z^{\ast },t^{\ast },\{g_{b_{u}}^{\ast }\})$ be an optimal solution of (24) and suppose that (24e) is not active at the optimum, i.e., $\frac {|\mathbf {h}_{b,b_{u}}\mathbf {v}_{b_{u}}^{\ast }|^{2}}{G_{b_{u}}(\mathbf {v}^{\ast })+\sigma _{b_{u}}^{2}}>g_{b_{u}}^{\ast }$ for some *b*_*u*_. Then, we can scale down the transmit power for user *b*_*u*_ and achieve a new beamformer $\|\hat {\mathbf {v}}_{b_{u}}\|_{2}^{2}$ such that $\|\hat {\mathbf {v}}_{b_{u}}\|_{2}^{2}=\tau \|\mathbf {v}^{\ast }_{b_{u}}\|_{2}^{2}<\|\mathbf {v}^{\ast }_{b_{u}}\|_{2}^{2}$ for *τ*∈(0,1) while keeping the others remaining unchanged, i.e., $\hat {\mathbf {v}}_{b_{k}}=\mathbf {v}_{b_{k}}^{\ast }$ for all *b*_*k*_≠*b*_*u*_. By this way, we can achieve $\frac {|\mathbf {h}_{b,b_{u}}\hat {\mathbf {v}}_{b_{u}}|^{2}}{G_{b_{u}}(\hat {\mathbf {v}})+\sigma _{b_{u}}^{2}}>g_{b_{u}}^{\ast }$ for all *b*_*u*_ since interference power at all users has reduced. In addition, we have $\hat {t}=P_{\text {total}}(\hat {\mathbf {v}})<t^{\ast }=P_{\text {total}}(\mathbf {v}^{\ast })$. Consequently, we can find $\hat {\eta }=\frac {(z^{\ast })^{2}}{\hat {t}}>\eta ^{\ast }$. This contrasts to the fact that $\left (\mathbf {v}^{\ast },\eta ^{\ast },z^{\ast },t^{\ast },\left \{g_{b_{u}}^{\ast }\right \}\right)$ is the optimal solution. The same spirit is applied to the other constraints. This completes the proof.

## Footnotes

1 An equal number of users in each cell are assumed merely for lightening up the notations.

2 The algorithm frameworks provided in this paper can be straightforwardly extended to the case of joint transmission as well.

3 By nontractable, we mean that it cannot be reformulated as an equivalent convex program, or such a transformation is not known in the literature.

4 The EE fairness measure is calculated following the index defined in ([41], (1)). Particularly, let us denote $\boldsymbol {\eta }^{\ast }=[\eta _{1}^{\ast },\ldots \eta _{B}^{\ast }]$ as the achieved per-BS EEs after solving, e.g., WSEE problems (28), WPEE problem (33), or maxminEE (36). The fairness index is $\text {fairness} = \frac {\bigl (\sum _{b=1}^{B}\eta _{b}^{\ast }\bigr)^{2}}{B\sum _{b=1}^{B}(\eta _{b}^{\ast })^{2}}$.
